# Linking health lifestyle classes to blue-collar workers’ participation in worksite health promotion programs in freight transport: a latent class analysis

**DOI:** 10.1007/s00420-025-02175-2

**Published:** 2025-11-11

**Authors:** Marc A. W. Damen, Sarah I. Detaille, Luuk P. van Iperen, Josephine A. Engels, Annet H. De Lange

**Affiliations:** 1 Faculty of Psychology, Open Universiteit, Heerlen, The Netherlands; 2 School of Organization and Development, Research Group Occupation and Health, HAN University of Applied Sciences, Nijmegen, The Netherlands; 3 School of Organization and Development, Applied Psychology, HAN University of Applied Sciences, Nijmegen, The Netherlands; 4 School of Organization and Development, Research Group Human Capital Innovations, HAN University of Applied Sciences, Nijmegen, The Netherlands; 5 Department of Social Medicine, Maastricht University, Maastricht, The Netherlands; 6 Division Strategy & Change, Radboud University, Nijmegen, The Netherlands; 7 Faculty of Social Sciences, Hotel School of Management, University of Stavanger, Stavanger, Norway; 8 Department of Psychology, Norwegian University of Science and Technology, Trondheim, Norway; 9 Department of Psychology, University of Coruña, A Coruña, Spain

**Keywords:** Blue-collar workers, Worksite health promotion programs (WHPPs), Participation, Implementation, Transport, Latent class analysis

## Abstract

**Purpose:**

Worksite health promotion programs (WHPPs) are able to promote a healthier lifestyle for blue-collar workers in freight transport, yet their participation is generally low. This study aims to identify different health lifestyle classes among blue-collar workers in freight transport and investigate the relationship between class membership and WHPP participation.

**Methods:**

Data from 16,897 employees were obtained from an online health questionnaire as part of a sector induced WHPP (89.1% male, 71.0% blue-collar worker, *M*_age_ = 49.3 years (*SD* = 12.7)). A latent class analysis was conducted to identify health lifestyle classes. Classes for blue-collar and white-collar workers were compared. Characteristics of the blue-collar workers’ classes were examined together with the likelihood of WHPP participation.

**Results:**

For blue-collar workers, a 5-class solution provided the best fit. These classes were labeled: (1) “unhealthy diet” (14%), (2) “health promoting” (29%), (3) “lack of moderate physical activity” (31%), (4) “low physical activity” (15%), and (5) “sleep deprived” (11%). For white-collar workers, a 4-class solution provided the best fit, with three comparable classes and one “health compromising” class. Blue-collar workers of the “unhealthy diet” and “sleep deprived” class reported the lowest perceived health, and showed highest WHPP participation levels. “Low physical activity” class members reported unhealthy behaviors, yet showed lowest participation levels.

**Conclusions:**

These findings indicate that different lifestyle classes exist among blue-collar workers within the freight transport industry which can be linked to WHPP participation. Consequently, WHPPs and implementation strategies can be adjusted to serve existing classes among blue-collar workers within the industry, in order to enhance participation.

## Introduction

Compared to their white-collar counterparts, blue-collar workers exhibit lower life expectancy (Deeg et al. 2021; Katikireddi et al. 2017), increased prevalence of severe physical health complaints; and diminished work ability (Schreuder et al. 2008; Van den Borre and Deboosere 2018). For example, truck drivers, the largest segment of blue-collar workers in freight transport, are at heightened risk of developing chronic diseases including cardiovascular diseases, diabetes, and musculoskeletal disorders (Apostolopoulos et al. 2013; Guest et al. 2020; van der Beek 2012). Unhealthy lifestyle choices represent a significant contributing factor to the observed health disparities between blue-collar and other workers (Kelly et al. 2014; Petrovic et al. 2018; Väisanen et al. 2023). Specifically, truck drivers are more prone to engaging in detrimental behaviors such as smoking, consuming unhealthy diets, lack of physical activity, and poor sleep patterns (Guest et al. 2020; van der Beek 2012), ultimately impacting their health and overall work ability.

The Sector Institute for Transport and Logistics (STL), a collaborative endeavor between industry’s unions and employers' organizations in the Netherlands, offers a WHPP aimed at promoting a healthy lifestyle and improving general health and work ability among employees. Between 2014 and 2021, 19,564 workers (of which there were 12,422 blue-collar workers) in the sector completed a health assessment questionnaire as part of the WHPP. Of these workers, nearly 35% were identified as having a health risk including having a low work ability, or being obese. However, only 22% of the blue-collar workers with a health risk profile attended an initial consultation with a lifestyle coach, and less than half of these 22% participated in an offered follow-up intervention. These low participation levels can seriously hinder the impact of the WHPP, as previous research suggests that participation in interventions following health assessments are imperative for success (Soler et al. 2010). Increasing participation among transport workers in such interventions could improve the lifestyle and health of blue-collar workers in freight transport as a whole.

Tailoring WHPPs to the specific needs of blue-collar workers has been shown to enhance both their effectiveness (Ng et al. 2015; Puhkala et al. 2015; Virgara et al. 2024) and participation levels (Schaap et al. 2020; Wronska et al. 2022). Despite these promising outcomes, the relationship between specific lifestyle behaviors and WHPP participation among blue-collar workers remains underexplored. While sleep behaviors have been linked to WHPP participation, other single lifestyle behaviors such as physical activity, nutrition, or smoking have not (Damen et al. 2023), possibly due to subgroup variations within the population. Most research on factors influencing participation in WHPPs typically employs a variable-centered approach, examining the relationship between lifestyle behaviors and participation at a population level. However, a person-centered approach has the potential to reveal differences in participation rates among subgroups that exhibit similar lifestyle patterns. A person-centered approach may offer a more nuanced understanding of the variations in associations within distinct subgroups (Howard and Hoffman 2018).

Health Lifestyle Theory provides a compelling framework to address these variations. The theory posits that lifestyle behaviors cluster into specific patterns within individuals rather than occurring as independent choices (Cockerham 2005; Mollborn et al. 2021). While traditional class distinctions (e.g., white- vs. blue-collar) highlight structural differences, a health lifestyle perspective captures classes based on shared behaviors regardless of occupational status. This approach is particularly suited to identifying health behavior patterns that go beyond conventional class boundaries, and capturing underlying heterogeneity within and between social classes that might otherwise remain undetected in traditional class frameworks.

Indeed, prior research has identified distinct lifestyle behavior classes within populations (e.g., Collins and Lanza 2009; De Vries et al. 2008; Saint Onge and Krueger 2017). Importantly, lifestyle class membership has been associated with health outcomes (Kim et al. 2020), self-rated health (Patel and Moake 2023), and sustained participation in a lifestyle intervention (Ratz et al. 2021).

Understanding the heterogeneous distribution of lifestyle behaviors among blue-collar workers could provide insights for tailoring WHPPs to meet the specific needs of each lifestyle class. Such an approach may address multiple unhealthy behaviors simultaneously, as they tend to co-occur within classes. Integrated WHPPs that focus on clusters of relevant and related behaviors are not only more likely to succeed (Prochaska et al. 2008; Rhodes et al. 2016; Xia et al. 2020), but have also been found to engage blue-collar workers more effectively (Hunt et al. 2005).

### Aim of the study

In order to enhance the impact of current WHPPs in the freight transport industry, this study aims to identify and understand the different lifestyle classes present within the industry and how they may impact participation levels in WHPPs. The following research questions are addressed: Which distinct latent classes, if any, based on lifestyle characteristics, can be identified among blue-collar workers in the freight transport industry? What differences in number and response patterns, if any, are apparent between blue-collar and white-collar workers? What are demographic, work, and health characteristics of the classes that were found for blue-collar workers? And how do these classes relate to blue-collar workers’ WHPP participation? By gaining a better understanding of these classes, policy makers and HR professionals within transport companies can tailor their strategies to target specific segments of the workforce that may have not currently been participating in WHPPs.

### Methods

#### Sample and procedure

A secondary analysis was conducted on data obtained from an online health assessment that was part of the WHPP provided by STL, and through attendance registration of an offered consultation with a lifestyle coach. Participation in the WHPP was voluntary and confidential. While some organizations actively promoted the program, others offered no endorsement. Nevertheless, all employees of affiliated companies had free access to the online health assessment via the STL website. From 2014 to 2021, a total of 19,564 workers in the industry completed the health assessment. All participants provided informed consent. The study was carried out in compliance with the Declaration of Helsinki, and received approval from the Ethics Committee of HAN University of Applied Sciences (ECO 197.09/20, 4 September 2020).

Out of the initial sample, 109 respondents were excluded due to zero response variance on at least three questionnaire scales, 2,134 were excluded for not having blue-, or white-collar jobs, and 424 were excluded for missing data on all variables, resulting in a sample of 16,897 respondents. Chi-square tests and Levene’s t-test revealed that excluded respondents were younger (*F* = 105.579, *p* < 0.001, *M*_dif_ = -3.4 years), more likely to be blue-collar worker (*χ*^*2*^ = 18.567, *p* < 0.001), and employed in organizations with fewer than 10 employees (*χ*^*2*^ = 29.441, *p* < 0.001). No significant differences were observed in terms of gender and irregular working hours.

**Fig. 1:**
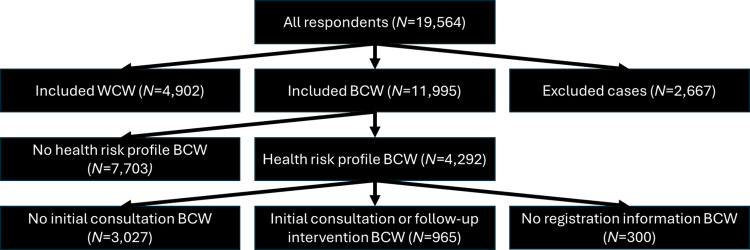
Flow of participants through the study. Note: BCW = blue-collar workers, WCW = white-collar workers

Workers were identified as “at-risk” based on their current physical and mental health status, lifestyle, and willingness to make changes. They were offered an initial consultation and a follow-up intervention. The follow-up intervention involved sessions with a lifestyle coach or dietician targeting key risk behaviors such as smoking cessation, weight loss, or stress reduction. For the analysis, data from all included blue-collar workers (*N* = 11,995) and white-collar workers (*N* = 4,902) were utilized for the three first research questions on latent classes. Only data from blue-collar workers who were offered a consultation or follow-up intervention (*N* = 3,992) were used for the final research question on class membership and participation. The flow of participants through the study is depicted in Fig. 1.

### Measures

*Lifestyle behavior and self-rating.* Ten indicators were utilized for the latent class analysis. For the categorization, recommendations were used from the Health Council of the Netherlands (Health Council of the Netherlands 2006, 2017). *Alcohol consumption* was assessed by asking about the frequency of drinking alcohol per week (ranging from “zero” to “every day”) and the average number of glasses consumed per day (ranging from “one” to “more than six”. Respondents were categorized as “not drinking”, “moderate” (1 to 7 glasses per week), “high” (8 to 14 glasses per week for women and 8 to 21 glasses for men) or “excessive” (15 glasses or more for women and 22 glasses or more for men). *Smoking* was assessed by asking respondents if they currently smoked with response options of “no”, “sometimes”, or “daily”. *Nutrition (vegetable intake and fruit intake)* was assessed by asking about the frequency of vegetable and fruit consumption per week (ranging from “zero” to “every day”), as well as the average daily intake in grams for vegetables and number of pieces for fruits (ranging from “less than 100 g” to “more than 400 g” for vegetables, and from “less than one” to “four or more” for fruits). Respondents were classified as “recommendation-adherent” if they consumed an average of 200 g of vegetables per day or two pieces of fruit per day. Respondents could also be classified as “just below recommendation” (no less than 150 g of vegetables per day or 1.5 pieces of fruit per day on average), “below recommendation” (no less than 100 g of vegetables per day or 1 piece of fruit per day) or “far below recommendation” (less than 100 g of vegetables per day or less than 1 piece of fruit per day). *Physical activity (moderate PA and vigorous PA)* was assessed by having respondents report the frequency of engagement in both moderate PA and vigorous PA per week: “never”, “1 or 2 times”, “3 or 4 times”, or “5 times or more”. For moderate PA, 5 times a week, and for vigorous PA, at least once a week vigorous PA was considered recommendation-adherent. *Sleep* was assessed by asking respondents about their average hours of sleep per night (“less than 6 h”, “6 to 8 h”, or “more than 8 h”). Respondents were classified as “insufficient sleep” when they slept less than 6 h per night, or as “sufficient sleep” when they slept more than 6 h. *Self-rating (self-rated nutrition, self-rated PA, and self-rated sleep)* involved respondents rating their own dietary habits, physical activity levels, and sleep quality as sufficiently healthy, with options of “yes” or “no”. The questionnaire that was used in this study has not been tested for reliability and validity. Comparable international screening instruments have shown moderate to good reliability and validity, although some concerns have been expressed regarding the external validity of PA self-assessment when tested against objective, device based measures (Bully 2016; Tcymbal et al. 2024).

*Socio-demographic characteristics.* Respondents were asked to state their *gender*, *age* (measured in years), and *education level* (ranging from “no education” to “master”). Education level was categorized according to the International Standard Classification of Education 2011 (ISCED) levels (UNESCO 2012), with levels 0, 1, and 2 classified as “basic education”, levels 3, 4, and 5 as “practical education”; and levels 6, 7, and 8 as “theoretical education”. *Occupation* was assessed by allowing respondents to select from nine most common occupations in freight transport. Respondents were categorized into *job type* based on the International Standard Classification of Occupations (ISCO) (International Labour Organization 2012). Truck drivers, warehouse workers, technicians, cleaners, crane operators, and movers (ISCO codes 1–5) were categorized as “blue-collar”. Office workers, planners, and managers (ISCO codes 6–9) were categorized as “white-collar”. Respondents who selected “other” as their occupation were classified as “other”.

*Work characteristics.* Subjects were asked to indicate if they worked in shifts or had *irregular working hours*, and to estimate the *size of their company*. Company size estimates falling below 1 or above 1.6 million were treated as missing data. Estimates within the defined range were categorized into “micro” (1–9 employees), “small” (10–49 employees), “medium” (50–249 employees), or “large” (≥ 250 employees) companies, in accordance with European Union definitions (see European Commission (2003)).

(*Self-rated) health*. Respondents were asked if they had ever been diagnosed with specific *chronic diseases*. The list, derived from the Work Ability Index (WAI) (Tuomi et al. 2003), contained 13 predefined conditions and the possibility to state whether they had been diagnosed with a chronic disease that was not on the list. Respondents were categorized as having “no chronic disease” or “one or more chronic disease”. Additionally, respondents *rated their overall health* on a 5 point Likert scale (“poor” (= 1) to “excellent” (= 5)), derived from the Short Form 12 (SF-12) (Ware et al. 1996), and were classified as “reporting poor or moderate health” or “reporting good to excellent health”. This single-item indicator has been used widely in epidemiologic research, and have shown strong predictive validity for mortality (Schnittker and Bacak 2014) and strong correlations with physical symptoms rather than manifest diseases (Eriksson et al. 2001).

*Participation.* Participation was measured through the coaches’ registration of attendance at the first consultation session, resulting in two possible outcomes: “participation” or “no participation”. Registration data were available only for “at risk” respondents with recorded attendance yielding a sample of 3,992 blue-collar workers.

### Statistical analysis

Descriptive analyses were conducted using SPSS, version 27. Means, standard deviations, frequencies, and zero-order correlations were calculated.

Latent class analysis (LCA) was performed using MPlus (version 8.11) to identify latent groups in the study population based on their response patterns to the ten lifestyle behavior and self-rating indicators. Separate LCAs were conducted for blue-collar and white-collar workers to compare class solution between the two groups. LCA was chosen for its model-based approach, fit statistics generation, and statistical inference capabilities, making it a more robust method compared to cluster analysis (Sinha et al. 2021; Weller et al. 2020). The sample size of this study exceeded the recommended minimum of *N* = 500 for LCA (Nylund et al. 2007; Spurk et al. 2020).

The number of starting values was set at 20,000 with 2,500 iterations. The 2,000 best fits were used for comparing results. The optimal number of latent classes was determined by sequentially comparing the model fit of each class solution while incrementally increasing the number of classes beyond the first, from two up to eight classes. The fit of each class solution was examined using a variety of indicators, including statistical criteria, parsimony, and interpretability (Collins and Lanza 2009; Sinha et al. 2021; Weller et al. 2020). To narrow down the number of possible solutions, Akaike Information Criterion (AIC), Consistent Akaike Information Criterion (CAIC), Bayesian Information Criterion (BIC), and Sample-Adjusted BIC (SABIC) were utilized, with lower scores implying better fit for all four criteria (Collins and Lanza 2009). When working with large sample sizes, BIC is considered as most robust and superior to the other three indices (Nylund et al. 2007; Sinha et al. 2021). The model was then tested using Lo-Mendell-Rubin Likelihood Ratio Test (LMR-LRT), and Bootstrap Likelihood Ratio Test (BLRT). Both provide statistical tests of the improvement in model fit when adding an additional class, with BLRT considered more robust for evaluating model fit (Nylund et al. 2007). Additional considerations for determining class number included entropy (ideally above 0.80), class size (preferably all above 5–8%), average and lowest class probability (preferably above 0.80), class interpretability, and uniqueness (Collins and Lanza 2009; Ferguson et al. 2020; Nylund-Gibson and Choi 2018; Sinha et al. 2021; Spurk et al. 2020).

To analyze class composition among the blue-collar workers, demographic, work, and health variables were included while keeping the latent class parameters fixed using the 3-step method (Asparouhov and Muthén 2014). The relationship between latent class membership and blue-collar workers’ participation in WHPPs was examined through the Bolck-Croon-Hagenaars (BCH) approach (Bolck et al. 2004), currently the preferred method for binary outcomes (Asparouhov and Muthén 2021; Nylund-Gibson et al. 2019). A semi-constrained model was tested with indicator means for each class held equal.

## Results

### Descriptives

In this study, 89.1% of participants were male (*N* = 15,052), 70.1% were employed in blue-collar occupations (*N* = 11,995), and 42.7% had a basic level of education (*N* = 7,223). The mean age of the sample was 49.3 years (*SD* = 12.7). These proportions align with those typically seen in the industry, although the blue-collar/white-collar worker ratio in our sample (2.4:1) differed from that typically found in the sector (4.1:1), Moreover, the mean age in our sample (49.3) was higher than the industry average (42.7 years (STL 2024)).

Regarding recommendation-adherent health behaviors, 80% of the respondents reported sleeping 6 h or more per night. For PA, 23% engaged in moderate PA at least 5 times per week. Additionally, 67% participated in vigorous PA at least once a week. in terms of nutrition, 10% consumed an average of at least 200 g of vegetables daily, and 17% ate an average of at least 2 pieces of fruit each day. Furthermore, 73% adhered to the guideline of consuming no more than an average of seven glasses of alcohol per week, and 70% reported not smoking.

Correlations among indicators were assessed pair-wise using the Spearman’s correlation coefficient, revealing the highest correlation between moderate PA and vigorous PA for both the blue-collar worker (*ρ* = 0.451; *p* < 0.001) as the white-collar worker group (*ρ* = 0.464; *p* < 0.001). This falls within an acceptable range, according to previous research (Sinha et al. 2021).

### Latent class analysis

Fit statistics and decision criteria of the latent class analyses are presented in Table 1 for blue-collar workers and in Table 2 for white-collar workers. For both groups, SABIC and BLRT showed that adding classes perpetually kept improving the model. LMR-LRT, entropy and elbow-plotting of SABIC and AIC favored the 5-class solution for blue-collar workers, and a 4-class solution for white-collar workers. Furthermore, lowest class membership probability dropped from 78% in the 5-class solution to 75% in the 6-class solution, showing more robustness of the 5-class solution for blue-collar workers. For white-collar workers, lowest class membership probability dropped from 79% in the 4-class solution to 69% in the 5-class solution. To conclude, all indicators pointed either towards the maximum number of classes or the 5-class solution for blue-collar workers and the 4-class solution for white-collar workers. As more complex models with many small classes are less generalizable (Sinha et al. 2021), the most parsimonious solutions (i.e. the 5 and 4 class solution) were chosen. Moreover, these solutions contained classes that were interpretable and sufficiently distinct from one another.

**Table 1:** Fit statistics and decision criteria for all class solutions for blue-collar workers

	NFP	LL	AIC	BIC	SABIC	CAIC	LMR-LRT	BLRT	Entropy	LCP
2 class solution	43	-107,122	214,330	214,647	214,511	214,647	< 0.001*	< 0.001*	0.584	0.871
3 class solution	65	-106,154	212,438	212,918	212,711	212,918	< 0.001*	< 0.001*	0.633	0.819
4 class solution	87	-105,416	211,005	211,649	211,372	211,649	< 0.001*	< 0.001*	0.699	0.811
**5 class solution**	**109**	**-104,879**	**209,977**	**210,782**	**210,436**	**210,782**	< 0.001*	< 0.001*	**0.699**	**0.781**
6 class solution	131	-104,574	209,410	210,379	209,962	210,379	< 0.001*	< 0.001*	0.670	0.749
7 class solution	153	-104,323	208,952	210,083	209,597	210,083	< 0.001*	< 0.001*	0.653	0.635
8 class solution	175	-104,126	208,602	209,895	209,339	209,895	0.817	< 0.001*	0.659	0.666

N = 11,995; NFP = number of free parameters; LL = loglikelihood; BIC = Bayesian information criterion; SABIC = sample-size adjusted BIC; CAIC = consistent AIC; LMR-LRT = p-value of the Lo-Mendell-Rubin adjusted likelihood ratio Test; BLRT = p-value of the bootstrapped likelihood ratio test; LCP = lowest class probability; * p < 0.05; final class solution in bold script

**Table 2:** Fit statistics and decision criteria for all class solutions for white-collar workers

	NFP	LL	AIC	BIC	SABIC	CAIC	LMR-LRT	BLRT	Entropy	LCP
2 class solution	43	-41,441	82,969	83,248	83,112	83,248	< 0.001*	< 0.001*	0.678	0.901
3 class solution	65	-41,115	82,360	82,782	82,575	82,782	0.382	< 0.001*	0.703	0.818
**4 class solution**	**87**	**-40,852**	**81,878**	**82,443**	**82,166**	**82,443**	**0.129**	< 0.001*	**0.703**	**0.790**
5 class solution	109	-40,685	81,587	82,295	81,949	82,295	0.023*	< 0.001*	0.665	0.685
6 class solution	131	-40,522	81,307	82,158	81,742	82,158	< 0.001*	< 0.001*	0.731	0.704
7 class solution	153	-40,418	81,142	82,136	81,650	82,136	0.760	< 0.001*	0.732	0.654
8 class solution	175	-40,344	81,039	82,176	81,620	82,176	0.760	< 0.001*	0.745	0.695

N = 4,902; NFP = number of free parameters; LL = loglikelihood; BIC = Bayesian information criterion; SABIC = sample-size adjusted BIC; CAIC = consistent AIC; LMR-LRT = p-value of the Lo-Mendell-Rubin adjusted likelihood ratio Test; BLRT = p-value of the bootstrapped likelihood ratio test; LCP = lowest class probability; * p < 0.05; final class solution in bold script

Indicator probability scores for each of the classes are detailed in Tables 3 and 4. For blue-collar workers, five classes were identified: one concordant health promoting class, two concordant health compromising classes (“unhealthy diet” and “low physical activity”) and two discordant classes (“sleep deprived” and “lack of moderate physical activity”). Class proportions in the upper row are based on the estimated model. For white-collar workers, four classes were identified: one concordant “health promoting” class, one concordant health compromising class, and two discordant classes (“sleep deprived” and “lack of moderate physical activity”).

**Table 3:** Proportions for blue-collar workers’ lifestyle behaviors and self-rating by classes (N = 11,995)

	Overall	Class 1 (14.2%)	Class 2 (28.5%)	Class 3 (31.4%)	Class 4 (15.2%)	Class 5 (10.7%)
*Alcohol intake*						
Not drinking	0.16	0.17	0.18	0.13	0.18	0.17
Moderate (1 glass per day)	**0.55**	**0.52**	**0.54**	**0.58**	**0.54**	**0.56**
High (1 to 2 (women) or 1 to 3 (men) glasses per day)	0.26	0.27	0.25	0.27	0.24	0.25
Excessive (more than 2 (women) or 3 (men) glasses a day)	0.03	0.04	0.02	0.02	0.03	0.02
*Smoking*						
Not smoking	**0.68**	**0.57**	**0.74**	**0.70**	**0.61**	**0.71**
Sometimes	0.07	0.07	0.07	0.07	0.07	0.07
Daily	0.25	0.36	0.20	0.22	0.32	0.22
*Self-rated nutrition*						
Sufficient	**0.85**	0.24	**0.98**	**0.96**	**0.97**	**0.90**
Insufficient	0.15	**0.77**	0.03	0.04	0.03	0.10
*Vegetable intake*						
Recommendation-adherent	0.09	0.00	0.15	0.08	0.09	0.12
Just below recommendation	0.23	0.04	0.31	0.24	0.24	0.26
Below recommendation	**0.41**	0.24	**0.39**	**0.48**	**0.44**	**0.44**
Far below recommendation	0.27	**0.73**	0.16	0.20	0.22	0.18
*Fruit intake*						
Recommendation-adherent	0.16	0.04	0.23	0.17	0.13	0.21
Just below recommendation	0.13	0.05	0.18	0.14	0.11	0.14
Below recommendation	0.26	0.19	0.26	0.28	0.24	0.27
Far below recommendation	**0.45**	**0.72**	**0.33**	**0.41**	**0.52**	**0.37**
*Self-rated PA*						
Sufficient	**0.63**	0.26	**0.93**	**0.63**	0.47	**0.60**
Insufficient	0.37	**0.74**	0.07	0.37	**0.53**	0.40
*Moderate PA*						
>5 times a week	0.24	0.13	**0.58**	0.04	0.08	0.31
3–4 times a week	0.22	0.14	0.37	0.20	0.01	0.26
1–2 times a week	**0.38**	**0.48**	0.02	**0.71**	0.29	**0.36**
Never	0.16	0.26	0.03	0.05	**0.63**	0.08
*Intensive PA*						
>5 times a week	0.07	0.05	0.19	0.01	0.00	0.11
3–4 times a week	0.15	0.08	**0.36**	0.06	0.00	0.17
1–2 times a week	**0.41**	0.35	0.32	**0.70**	0.02	**0.45**
Never	0.36	**0.52**	0.14	0.23	**0.98**	0.27
*Self-rated sleep*						
Sufficient	**0.68**	0.48	**0.85**	**0.84**	**0.76**	0.00
Insufficient	0.32	**0.52**	0.15	0.16	0.24	**1.00**
Amount of sleep						
>6 h	**0.77**	**0.69**	**0.89**	**0.93**	**0.80**	0.10
< 6 h	0.23	0.32	0.11	0.07	0.20	**0.90**

Most common response category per class in bold script

**Table 4:** Proportions for white-collar workers’ lifestyle behaviors and self-rating by classes (N = 4,902)

	Overall	Class 1 (17.0%)	Class 2 (38.5%)	Class 3 (33.4%)	Class 4 (11.1%)
*Alcohol intake*					
Not drinking	0.14	0.18	0.13	0.11	0.19
Moderate (1 glass per day)	**0.61**	**0.59**	**0.64**	**0.61**	**0.57**
High (1 to 2 (women) or 1 to 3 (men) glasses per day	0.23	0.21	0.22	0.27	0.21
Excessive (more than 2 (women) or 3 (men) glasses per day)	0.02	0.02	0.01	0.02	0.03
*Smoking*					
Not smoking	**0.76**	**0.65**	**0.83**	**0.74**	**0.73**
Sometimes	0.07	0.07	0.07	0.08	0.09
Daily	0.17	0.27	0.10	0.19	0.19
*Self-rated nutrition*					
Sufficient	**0.90**	**0.79**	**0.97**	**0.90**	**0.82**
Insufficient	0.10	0.21	0.03	0.10	0.18
*Vegetable intake*					
Recommendation-adherent	0.12	0.08	0.18	0.07	0.14
Just below recommendation	0.23	0.20	0.28	0.20	0.22
Below recommendation	**0.43**	**0.40**	**0.39**	**0.49**	**0.42**
Far below recommendation	0.22	0.31	0.15	0.24	0.22
*Fruit intake*					
Recommendation-adherent	0.18	0.11	0.26	0.13	0.18
Just below recommendation	0.16	0.12	0.18	0.14	0.16
Below recommendation	0.28	0.25	**0.30**	0.29	0.24
Far below recommendation	**0.38**	**0.53**	0.26	**0.44**	**0.43**
*Self-rated PA*					
Sufficient	**0.58**	0.15	**0.93**	0.43	0.48
Insufficient	0.42	**0.85**	0.07	**0.57**	**0.52**
*Moderate PA*					
>5 times a week	0.22	0.05	0.43	0.06	0.23
3–4 times a week	0.30	0.05	**0.48**	0.21	0.30
1–2 times a week	**0.38**	0.39	0.08	**0.71**	**0.43**
Never	0.10	**0.50**	0.02	0.02	0.04
*Intensive PA*					
>5 times a week	0.05	0.00	0.12	0.00	0.06
3–4 times a week	0.21	0.00	**0.47**	0.03	0.17
1–2 times a week	**0.47**	0.10	0.32	**0.81**	**0.55**
Never	0.27	**0.90**	0.10	0.16	0.21
*Self-rated sleep*					
Sufficient	**0.72**	**0.66**	**0.85**	**0.83**	0.00
Insufficient	0.28	0.34	0.15	0.17	**1.00**
*Amount of sleep*					
>6 h	**0.86**	**0.83**	**0.94**	**0.97**	0.31
< 6 h	0.14	0.17	0.06	0.03	**0.69**

Most given response category per class in bold script

**Fig. 2:**
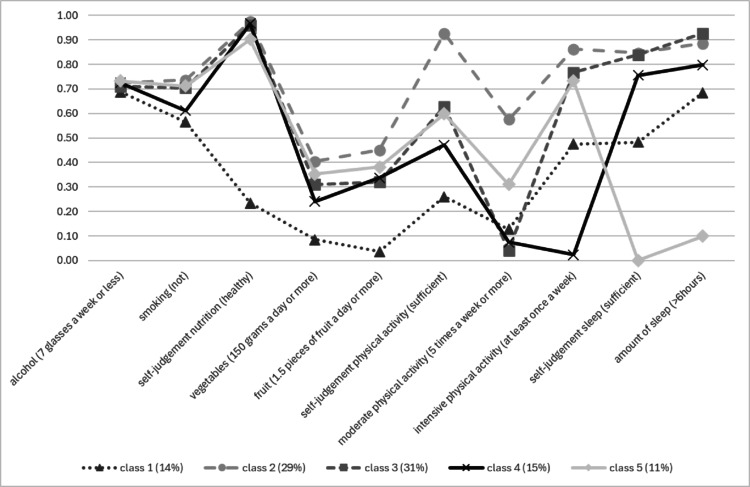
Proportions of health lifestyle behavior probabilities per class for blue-collar workers (N = 11,995)

**Fig. 3:**
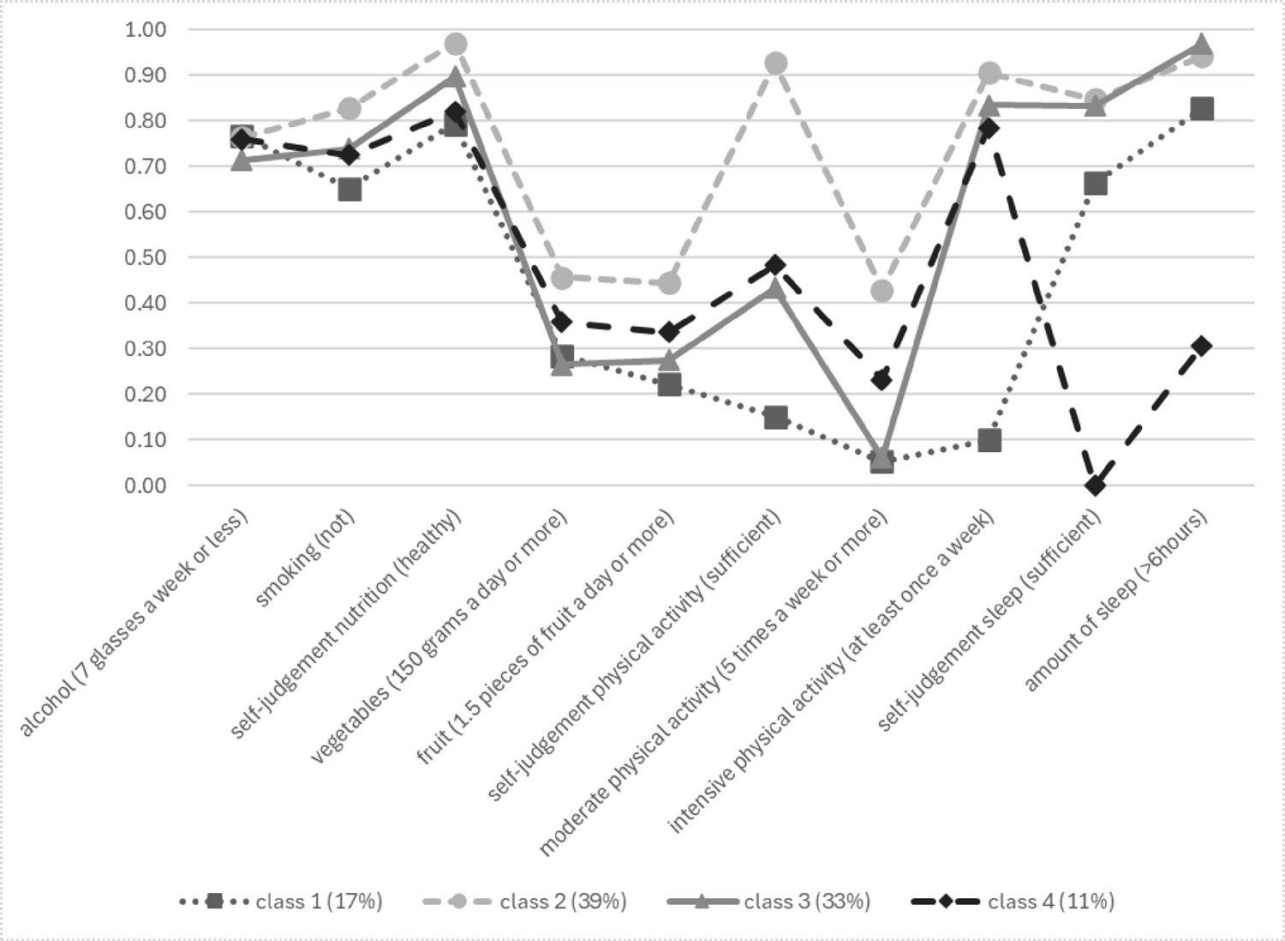
Proportions of health lifestyle behavior probabilities per class for white-collar workers (N = 4,902)

Figure 2 presents the probability scores for healthy lifestyle behaviors across latent classes for blue-collar workers, while Fig. 3 displays the corresponding scores for white-collar workers. To enhance interpretability, all indicators were dichotomized for visualization purposes. When adherence to specific recommendations did not clearly differentiate between classes, the categories 'just below recommendation' and 'recommendation-adherent' were combined. For instance, the indicator 'consuming at least 150 g of vegetables per day' provided clearer class differentiation than 'consuming at least 200 g per day', as adherence to the latter was low even within the healthiest class.

In Table 5, the proportions of several demographic, work, and health characteristics for each blue-collar worker lifestyle class are presented. Chi-square and *p*-values are given in the first column. All characteristics, except gender, were significantly related to class membership. In Table 6, the results of the BCH analysis are presented, showing the likelihood of WHPP participation for each blue-collar worker lifestyle class. Chi-square and *p*-values are given in the first column. Characteristics of the five classes and their relationship with WHPP participation are described below.

**Table 5:** Proportions for blue-collar workers’ sociodemographic, work and health characteristics by health lifestyle class (N = 11,995)

	All blue-collar workers	Class 1	Class 2	Class 3	Class 4	Class 5
*Gender (χ*^*2*^ = *4.303; p* = *0.367)*						
Male	0.97	0.96	0.96	0.97	0.97	0.96
Female	0.03	0.04	0.04	0.03	0.03	0.04
*Age (mean) (χ*^*2*^ = *254.682, p* < *0.001)*						
15–30	0.09	0.14	0.15	0.06	0.04	0.05
31–50	0.35	0.46	0.30	0.34	0.29	0.38
51–70	0.56	0.40	0.55	0.61	0.67	0.57
*Education level (χ*^*2*^ = *45.450, p* < *0.001)*						
ISCED level 1–3	0.51	0.49	0.49	0.51	0.59	0.51
ISCED level 4–6	0.45	0.48	0.47	0.45	0.38	0.46
ISCED level 7–9	0.04	0.03	0.05	0.04	0.03	0.04
*Job type (χ*^*2*^ = *239.868; p* < *0.001)*						
Driver	0.83	0.90	0.74	0.87	0.88	0.85
Warehouse worker	0.10	0.08	0.16	0.08	0.07	0.10
Technician	0.04	0.01	0.06	0.03	0.03	0.02
Cleaner, crane operator or mover	0.03	0.01	0.04	0.02	0.03	0.03
*Irregular working hours (χ*^*2*^ = *163.631, p* < *0.001)*						
No	0.27	0.17	0.35	0.28	0.27	0.20
Yes	0.73	0.83	0.65	0.72	0.73	0.80
*Company size (χ*^*2*^ = *44.343, p* < *0.001)*						
Micro (1–9)	0.03	0.02	0.03	0.03	0.03	0.04
Small (9–49)	0.16	0.17	0.17	0.15	0.15	0.14
Medium (50–249)	0.46	0.49	0.43	0.48	0.48	0.41
Large (>250)	0.35	0.32	0.37	0.33	0.35	0.42
*Lockdown (χ*^*2*^ = *40.389; p* < *0.001)*						
Pre-lockdown	0.91	0.94	0.88	0.92	0.93	0.92
Lockdown	0.09	0.06	0.12	0.08	0.07	0.09
*Self-rated health (X*^*2*^ = *350.805; p* < *0.001)*						
Good to excellent	0.88	0.74	0.94	0.93	0.88	0.76
Poor or moderate	0.12	0.27	0.06	0.07	0.12	0.24
*Number of chronic illnesses (χ*^*2*^ = *227.987; p* < *0.001)*						
No chronic illness	0.36	0.27	0.43	0.39	0.35	0.20
One or more	0.64	0.73	0.57	0.61	0.65	0.80

**Table 6:** Probability scores for participation outcomes by health lifestyle class (N = 3,992)

	All blue-collar workers	Class 1	Class 2	Class 3	Class 4	Class 5
*WHPP participation (χ*^*2*^ = *26.202, p* < *0.001)*						
No	0.76	0.71	0.80	0.78	0.80	0.69
Yes	0.24	0.29	0.20	0.22	0.20	0.31

*Class 1 (14.2%): concordant health-compromising: unhealthy diet:* The eating habits of members in this class do not meet health recommendations. They are more likely to smoke and less likely to exercise regularly or get enough sleep compared to the average worker. Members of this class are more likely than members of other classes to be under 30, have irregular working hours, and work in medium-sized companies. They are more likely to report poor to moderate health, have chronic illnesses, and participate in WHPPs.

*Class 2 (28.5%): concordant health-promoting*^1^*:* Members of class 2 tend to adhere more closely to health recommendations than members of other classes. They have lower rates of smoking and better sleep habits. However, many in this class do not meet the recommended intake for vegetables, fruits, and moderate PA, while perceiving their habits as healthy. Nonetheless, they outperform other classes in these areas. They are more likely than other workers to be under 30, theoretically educated, have regular working hours, work as a warehouse worker or technician, and to work in large companies. They are more likely to have no chronic illnesses, to report good to excellent health, and are less likely to participate in WHPPs.

*Class 3 (31.4%): discordant: lack of moderate physical activity:* Most notable in this class, is the lack of engagement in moderate PA, possibly indicating a sedentary lifestyle. However, members of this class engage in vigorous PA once or twice a week. They tend to sleep well, while scoring average on other indicators. Members of this class are more likely to be over 50 years old, have no chronic illness, and to report good to excellent health. They are less likely to participate in WHPPs.

*Class 4 (15.2%): concordant health-compromising: low physical activity:* Class 4 members lack vigorous PA and engage in minimal PA overall. They are more likely to smoke and have poor eating habits, while perceiving their eating habits as healthy. Members of this class are more likely to be over 50, and have basic education. Additionally, they show an average likelihood of having chronic illnesses, and reporting poor to moderate health. However, they are the least likely to participate in WHPPs.

*Class 5 (10.7%): discordant: sleep-deprived:* Members of class 5 all report not getting enough sleep, with most sleeping less than 6 h per night. They have average scores on other indicators. They are more likely to have irregular working hours, and work in large companies. They are more likely to have a chronic illness, and to report poor to moderate health. Furthermore, they are the most likely to participate in WHPPs.

## Discussion

### Discussion

In this study, we identified classes based on lifestyle behavior within the population of workers in the freight transport industry, and examined their association with participation in WHPPs. Among blue-collar workers, we identified five classes compared to four among white-collar workers.

Most LCA studies on lifestyle behaviors have found both health-promoting and health-compromising classes (e.g., De Vries et al. 2008; Saint Onge and Krueger 2017; Ratz et al. 2021; Collins and Lanza 2009; Xia et al. 2020). However, merely finding concordant classes may indicate that no classes exist, but a single population is spread out along a continuum (Sinha et al. 2021). As in other studies, next to concordant classes, we identified some discordant classes, but as De Vries et al. (2008) notes, it is difficult to compare these LCA results across studies, because found classes are highly dependent on chosen indicators and used techniques for performing the LCA. Furthermore, latent classes may be similar across populations, but show different response patterns. For example, in our study smoking and alcohol showed the least variation between classes, while other studies found larger variation (e.g. Saint Onge and Krueger (2017); Xia et al. (2020)). This may indicate that smoking is more equally distributed among blue-collar workers’ lifestyle classes in the Netherlands than in other populations. Moreover, these discrepancies in LCA results complicate the comparison of associations with outcome measures. For instance, Ratz et al. (2021) reported that, among older adults, individuals classified as socially inactive were most likely to discontinue participation in a physical activity intervention. The inclusion of social activity indicators alongside other health-related lifestyle behaviors in their analysis resulted in the identification of a class that, logically, did not emerge in our study.

We found that most blue-collar workers in our sample did not meet the recommended Dutch guidelines regarding PA and nutrition. For example, in our sample, only 10% met the recommended guidelines of eating more than 200 g of vegetables per day compared to the 27% reported in the general Dutch population (RIVM 2024), and 24% in our sample met the recommended guideline of at least 5 times a week engaging in moderate PA compared to 44% in the general Dutch population (RIVM 2024). This finding is in line with previous studies that found that blue-collar workers are more prone to engaging in unhealthy lifestyle behaviors (Guest et al. 2020; van der Beek 2012).

Even members of the health-promoting class exhibited behaviors that are not in line with recommended guidelines regarding fruits and vegetable intake, and moderate PA. Despite this, nearly all members of this class (98%) rated their own diet and physical activity as healthy, while in reality, fewer than 60% actually met the associated recommendations. This misperception may help explain their lower participation rates in workplace health promotion programs (WHPPs). As previous research has shown, not recognizing the need to improve one’s lifestyle can be a significant barrier to engaging in such programs (Smit et al. 2023).

Our finding that members of the “unhealthy diet” and the “sleep deprived” class were more likely to have irregular working hours, is in line with previous studies that found that shift workers show poorer quality diets, and poor quality and quantity of sleep (Hulsegge et al. 2021; Nea et al. 2015). Similarly, our finding that members of the “low PA” class were more likely to be older, corresponds with previous studies showing decrease in PA levels with age (Sun et al. 2013). Previous studies have found mixed results regarding the impact of lockdown measures on lifestyle (Bennett et al. 2021; Rubio-Tomás et al. 2022; Stockwell et al. 2021). Although the lockdown measures did not impact the class solution we found, we did find that during COVID lockdown, workers were more likely to be a member of the health promoting class. In particular for truck drivers, lockdown measures may have had stimulating effects on preparing homemade meals and walking during lunch breaks, due to closed truck stops and restricted access to their clients’ building.

In this study, WHPP participation varied from 19.9% to 30.6% across different classes. Members of the “sleep deprived” class were 1.39 times more likely to participate than other blue-collar workers, which seems substantial, compared to other calculated odds ratio’s for WHPP participation (see for example Robroek et al. (2009)). Previous research supports the idea that insufficient sleep is associated with increased participation in WHPPs among blue-collar workers, possibly caused by a stronger need for assistance in health behavior change, as sleep deficit is associated with obesity (Sorensen et al. 2009). Additionally, while unhealthy eating habits were not previously associated with WHPP participation (Hunt et al. 2010; Sorensen et al. 2010), our findings suggest that members in the "unhealthy diet" class are 1.40 times more likely to participate, potentially indicating the influence of overall lifestyle on participation.

Contrary to previous studies that found workers with healthy behaviors more likely to participate in WHPPs (Abraham et al. 2011; Groeneveld et al. 2009; Hall et al. 2017; Jørgensen et al. 2015), our study shows that blue-collar workers in the health promoting class are less likely to participate. Conversely, members of the two classes reporting the lowest perceived health are more inclined to participate. These findings imply that blue-collar workers may prioritize health differently than their counterparts, potentially feeling less motivated to modify their lifestyle habits when they perceive themselves to be healthy, as supported by previous research (Bukman et al. 2014; Wardle and Steptoe 2003).

### Theoretical implications

In line with health lifestyle theory, our findings suggest that lifestyle choices among blue-collar workers in freight transport tend to cluster into specific patterns. Notably, we identified discordant classes characterized by a mix of healthy and unhealthy behaviors. Consistent with lifestyle theory, which posits that lifestyle patterns are shaped by structural variables such as social class, age, and other demographic factors (Cockerham 2005), our study revealed correlations between these structural variables—except gender—and membership in specific lifestyle classes. However, establishing causal relationships will require more longitudinal research.

For blue-collar workers, two health compromising classes were identified in this study: one marked by unhealthy dietary patterns, and another by low PA. While previous research has shown dietary habits and PA levels often coexist (De Vries et al. 2008), our findings indicate a divergence in these behaviors among blue-collar workers. In contrast, the white-collar workers in our sample exhibited a single health-compromising class where unhealthy dietary habits and low PA co-occurred. This discrepancy may arise from distinct determinants influencing both behaviors for blue-collar workers, such as differences in nutritional knowledge or leisure time availability for PA, whereas for white-collar workers these behaviors may be more influenced by shared determinants, such as required intentional effort. These findings highlight the need for future research to explore how determinants of lifestyle behaviors and class membership vary between blue-collar and white-collar workers and their implications for designing targeted interventions.

### Practical implications

The classes we identified are associated with WHPP participation, providing a foundation for tailored WHPPs that address multiple relevant behaviors for each class, and for tailored implementation strategies.

The study results show that members of the “low physical activity” class exhibit unhealthy lifestyle behaviors and report relatively low self-rated health. They may be at risk of developing health problems but are the least inclined to participate in WHPPs. Programs targeting this class could focus on PA, nutrition and smoking. Since most of these workers do not yet experience health issues, communication could utilize gain frames, emphasizing the benefits of exercise and regular moderate PA. Given that this class includes relatively more older workers and workers with basic education, messaging should highlight the importance of PA at older age and be straightforward and easy to understand.

Furthermore, the results suggest that stimulating participation among members of the “health promoting” and “lack of moderate physical activity” classes could also be worthwhile. Although these individuals experience fewer health issues compared to other classes, most do not meet the recommended guidelines for nutrition and PA, yet perceive their behavior as healthy. This suggests a potential lack of awareness regarding their unhealthy lifestyle habits. In line with the transtheoretical model of behavior change (Prochaska and DiClemente 1983), recruitment strategies could focus on raising awareness of personal behavior to encourage progression from the precontemplation to the contemplation stage. Communication may include practical tips on healthy eating and incorporating PA into daily routines while on the road.

The two groups with the poorest health and self-rated health are the “unhealthy eating habits” and “sleep deprivation” classes. Programs for these classes could focus on nutrition, PA and smoking cessation for the “unhealthy eating habits” class, and on sleep, nutrition and PA for the “sleep deprivation” class. Although members of these classes are more likely to participate in WHPPs, irregular working hours may create barriers. Implementation strategies should therefore prioritize flexibility in location and scheduling, and communication should emphasize the importance of healthy nutrition and sleep habits, particularly for shift workers.

### Strengths and limitations

A key strength of this study was the use of LCA to identify lifestyle classes among blue-collar workers in freight transport. The large sample size provided increased power, and greater precision estimates of proportions and probabilities, and made it possible to conduct LCA with subgroups. Additionally, we were able to relate identified classes to WHPP participation, uncovering potential starting points for WHPP implementation.

However, several limitations should be noted. First, since the data we used were obtained from a large questionnaire containing 139 items, its length may have introduced selection bias, favoring white-collar workers over blue-collar workers (Damen et al. 2024). Another selection bias arose in analyzing the relationship between class membership and WHPP participation, as we only included respondents offered a follow-up intervention (~ 30% of the total group of respondents). The WHPP offer not only depended on severity of the health risk, but also on the willingness to change one’s lifestyle potentially affecting WHPP participation in some classes more than in others.

Second, we used self-report measures, which may introduce biases, such as recall bias and social desirability (e.g., underreporting alcohol use (Schell et al. 2021)). Additionally, the nature of some measures may have violated the local independence assumption in LCA. Indeed, certain indicators within some classes displayed high bivariate residual correlations (*z* >2.0). As Oberski (2016) points out, increasing the number of latent classes could reduce local dependency, but may not yield substantive new classes (e.g., adding a class for those answering in a socially desirable way). A follow-up study using objective measures, such as accelerometer data, could mitigate potential issues.

Third, we did not include potential covariates other than job type in our measurement and structural models. As demonstrated elsewhere, covariates can directly influence the indicators in the measurement model (Vermunt and Magidson 2021). By not taking covariates into account in the estimation of the latent class measurement model parameters, the existence of some classes independent of these covariates may be questionable. However, conditional independence tests showed no correlations above 0.4 between sociodemographic, work and health characteristics and indicators or outcomes. Future studies could incorporate covariates in the structural model and examine interaction effects between predicting variables (including class membership) for WHPP participation, preferably using longitudinal data.

Fourth, the study yielded relatively low overall entropy and class probability values. This could be attributed to its exploratory nature and the absence of available theory regarding lifestyle classes in this population. While some suggest that entropy and class probabilities should exceed 0.80 or even 0.90 (Weller et al. 2020), we accepted values of 0.70 and 0.78, respectively, given the sample size, overall fit and interpretability. Researchers applying stricter cut-offs might have chosen a different solution.

Fifth, our measures were based on Dutch lifestyle behavior recommendations, which may differ from those in other countries. For example, guidelines for alcohol use vary substantially (Kalinowski and Humphreys 2016). However, most guidelines in high income countries closely resemble Dutch standards (see for example US guidelines for PA (USDHHS 2018) or nutrition (USDA 2020)). It is important to note that substantial differences in guidelines might limit the transferability of our results.

Finally, while our aim was to provide policy makers and HR professionals with actionable insights for tailoring programs and implementation strategies we focused on initial WHPP participation as an outcome, whereas WHPP effectivity equally depends on starting the follow-up intervention, and continued and active participation or dose received. Future research could explore which classes are more likely to maintain participation and benefit from WHPPs.

## Conclusion

In this study, results were presented from a latent class analysis based on lifestyle characteristics conducted in the freight transport industry, with class membership related to WHPP participation. The following conclusions can be drawn:

1. Five lifestyle classes were identified among blue-collar workers: one concordant “health-promoting” class, two concordant health-compromising classes (“unhealthy diet” and “low physical activity”) and two discordant classes (“sleep-deprived” and “lack of moderate physical activity”);
2. Unhealthy dietary patterns and low levels of PA might be more closely linked among white-collar workers than among blue-collar workers;
3. Members of the “sleep-deprivation” class and the “unhealthy diet” class reported worst health and were most likely to participate in WHPPs;
4. Members of the “low physical activity” class are showing many health compromising behaviors, but are the least inclined to participate in WHPPs;
5. Most members of the “health promoting” class and “lack of moderate physical activity” class do not meet the recommended guidelines for nutrition and PA, yet they judge their own behavior as healthy.

These findings indicate that different lifestyle classes exist among blue-collar workers within the freight transport industry and these classes can be linked to WHPP participation. Consequently, WHPPs and implementation strategies should be adjusted to serve existing classes among blue-collar workers within the industry, in order to enhance participation.

## Data Availability

The data presented in this study are available on request from the corresponding author. The data are not publicly available due to privacy restrictions.

## Footnotes

1 Part of the data were collected during the COVID lockdown period. During this period blue-collar workers were more likely to be a member of this concordant health promoting class.

## References

[CR1] Abraham JM, Feldman R, Nyman JA, Barleen N (2011) What factors influence participation in an exercise-focused, employer-based wellness program? INQUIRY J Health Care Org Provision Financ 48(3):221–241. 10.5034/inquiryjrnl_48.03.01 10.5034/inquiryjrnl_48.03.01 22235547

[CR2] Apostolopoulos Y, Sönmez S, Shattell MM, Gonzales C, Fehrenbacher C (2013) Health survey of U.S. long-haul truck drivers: work environment, physical health, and healthcare access. Work 46(1):113–123. 10.3233/WOR-121553 23324711 10.3233/WOR-121553

[CR3] Asparouhov T, Muthén B (2014) Auxiliary variables in mixture modeling: three-step approaches using M plus. Struct Equ Model 21(3):329–341. 10.1080/10705511.2014.915181

[CR4] Asparouhov T, Muthén B (2021) Auxiliary variables in mixture modeling: using the BCH method in Mplus to estimate a distal outcome model and an arbitrary secondary model. In: Mplus web notes. https://www.statmodel.com/examples/webnotes/webnote21.pdf. Accessed 5 Dec 2024

[CR5] Bennett G, Young E, Butler I, Coe S (2021) The impact of lockdown during the COVID-19 outbreak on dietary habits in various population groups: a scoping review. Front Nutr 8:626432. 10.3389/fnut.2021.626432 33748175 10.3389/fnut.2021.626432 PMC7969646

[CR6] Bolck A, Croon M, Hagenaars J (2004) Estimating latent structure models with categorical variables: one-step versus three-step estimators. Polit Anal 12(1):3–27. 10.1093/pan/mph001

[CR78] Bukman AJ, Teuscher D, Feskens EJM, Baak MAv, Meershoek A, Renes RJ (2014) Perceptions on healthy eating, physical activity and lifestyle advice: opportunities for adapting lifestyle interventions to individuals with low socioeconomic status. BMC public health 14(1):1-11. 10.1186/1471-2458-14-1036 10.1186/1471-2458-14-1036 PMC4210550 25280579

[CR7] Bully P et al (2016) Metric properties of the “prescribe healthy life” screening questionnaire to detect healthy behaviors: a cross-sectional pilot study. BMC Public Health 16:1228. 10.1186/s12889-016-3898-8 27923356 10.1186/s12889-016-3898-8 PMC5142282

[CR8] Cockerham WC (2005) Health lifestyle theory and the convergence of agency and structure. J Health Soc Behav 46(1):51–67. 10.1177/002214650504600105 15869120 10.1177/002214650504600105

[CR9] Collins LM, Lanza ST (2009) Latent class and latent transition analysis: with applications in the social, behavioral, and health sciences. John Wiley & Sons Inc., Hoboken

[CR10] Damen MAW, Detaille SI, Engels JA, De Lange AH (2024) Perceived factors influencing blue-collar workers’ participation in worksite health promotion programs in freight transport: a qualitative investigation using the TDF and COM-B. Int J Environ Res Public Health. 10.3390/ijerph21010116 10.3390/ijerph21010116 PMC10815228 38276810

[CR11] Damen MAW, Detaille SI, Robroek SJW, Engels JA, de Lange AH (2023) Factors associated with blue-collar workers’ participation in worksite health promotion programs: a scoping literature review. Health Promot Int. 10.1093/heapro/daad052 10.1093/heapro/daad052 PMC10306361 37379570

[CR12] De Vries H et al (2008) Clusters of lifestyle behaviors: results from the Dutch SMILE study. Prev Med 46(3):203–208. 10.1016/j.ypmed.2007.08.005 17904212 10.1016/j.ypmed.2007.08.005

[CR13] Deeg DJH, De Tavernier W, de Breij S (2021) Occupation-based life expectancy: actuarial fairness in determining statutory retirement age. Front Sociol 6:675618. 10.3389/fsoc.2021.675618 34497844 10.3389/fsoc.2021.675618 PMC8419329

[CR14] Eriksson I, Undén A, Elofsson S (2001) Self-rated health. Comparisons between three different measures. Results from a population study. Int J Epidemiol 30(2):326–333. 10.1093/ije/30.2.326 11369738 10.1093/ije/30.2.326

[CR15] Commission E (2003) Definition of micro, small and medium-sized enterprises adopted by the commission. Off J Eur Union 46:39–41

[CR16] Ferguson SL, Moore GEW, Hull DM (2020) Finding latent groups in observed data: a primer on latent profile analysis in Mplus for applied researchers. Int J Behav Dev 44(5):458–468. 10.1177/0165025419881721

[CR17] Groeneveld IF, Proper KI, van der Beek AJ, Hildebrandt VH, van Mechelen W (2009) Factors associated with non-participation and drop-out in a lifestyle intervention for workers with an elevated risk of cardiovascular disease. Int J Behav Nutr Phys Act 6:80. 10.1186/1479-5868-6-80 19951417 10.1186/1479-5868-6-80 PMC3224927

[CR18] Guest AJ, Chen Y-L, Pearson N, King JA, Paine NJ, Clemes SA (2020) Cardiometabolic risk factors and mental health status among truck drivers: a systematic review. BMJ Open 10(10):e038993. 10.1136/bmjopen-2020-038993 10.1136/bmjopen-2020-038993 PMC7590350 33099498

[CR19] Hall JL, Kelly KM, Burmeister LF, Merchant JA (2017) Workforce characteristics and attitudes regarding participation in worksite wellness programs. Am J Health Promot 31(5):391–400. 10.4278/ajhp.140613-QUAN-283 26730552 10.4278/ajhp.140613-QUAN-283

[CR20] Health Council of the Netherlands (2006) Guidelines for a healthy diet 2006. Health Council of the Netherlands, The Hague, 2006; publication no. 2006/21E

[CR21] Health Council of the Netherlands (2017) Physical activity guidelines 2017. Health Council of the Netherlands, The Hague, 2017; publication no. 2017/08e

[CR22] Howard MC, Hoffman ME (2018) Variable-centered, person-centered, and person-specific approaches: where theory meets the method. Organ Res Methods 21(4):846–876. 10.1177/1094428117744021

[CR23] Hulsegge G, Proper KI, Loef B, Paagman H, Anema JR, van Mechelen W (2021) The mediating role of lifestyle in the relationship between shift work, obesity and diabetes. Int Arch Occup Environ Health 94(6):1287–1295. 10.1007/s00420-021-01662-6 33704584 10.1007/s00420-021-01662-6 PMC8292292

[CR24] Hunt MK, Harley AE, Stoddard AM, Lederman RI, MacArthur MJ, Sorensen G (2010) Elements of external validity of tools for health: an intervention for construction laborers. Am J Health Promot 24(5):e11–e20. 10.4278/ajhp.080721-QUAN-130 20569107 10.4278/ajhp.080721-QUAN-130

[CR25] Hunt MK et al (2005) Process evaluation of an integrated health promotion/occupational health model in WellWorks-2. Health Educ Behav 32(1):10–26. 10.1177/1090198104264216 15642751 10.1177/1090198104264216

[CR26] International Labour Organization (2012) International standard classification of occupations (ISCO-08) volume 1 structure, group definitions and correspondence tables. International Labour Office, Geneva

[CR27] Jørgensen MB, Villadsen E, Burr H, Mortensen OS, Holtermann A (2015) Does workplace health promotion in Denmark reach relevant target groups? Health Promot Int 30(2):318–327. 10.1093/heapro/dat041 23770769 10.1093/heapro/dat041

[CR28] Kalinowski A, Humphreys K (2016) Governmental standard drink definitions and low-risk alcohol consumption guidelines in 37 countries. Addiction 111(7):1293–1298. 10.1111/add.13341 27073140 10.1111/add.13341

[CR29] Katikireddi SV, Whitley E, Lewsey J, Gray L, Leyland AH (2017) Socioeconomic status as an effect modifier of alcohol consumption and harm: analysis of linked cohort data. Lancet Public Health 2(6):e267–e276. 10.1016/S2468-2667(17)30078-6 28626829 10.1016/S2468-2667(17)30078-6 PMC5463030

[CR30] Kelly IR, Dave DM, Sindelar JL, Gallo WT (2014) The impact of early occupational choice on health behaviors. Rev Econ Househ 12(4):737–770. 10.1007/s11150-012-9166-5 32863809 10.1007/s11150-012-9166-5 PMC7451157

[CR31] Kim S, Cho S, Nah E-H (2020) The patterns of lifestyle, metabolic status, and obesity among hypertensive Korean patients: a latent class analysis. Epidemiol Health. 10.4178/epih.e2020061 10.4178/epih.e2020061 PMC7871153 32882119

[CR32] Mollborn S, Lawrence EM, Saint Onge JM (2021) Contributions and challenges in health lifestyles research. J Health Soc Behav 62(3):388–403. 10.1177/0022146521997813 34528487 10.1177/0022146521997813 PMC8792463

[CR33] Nea FM, Kearney J, Livingstone MBE, Pourshahidi LK, Corish CA (2015) Dietary and lifestyle habits and the associated health risks in shift workers. Nutr Res Rev 28(2):143–166. 10.1017/S095442241500013X 26650243 10.1017/S095442241500013X

[CR34] Ng MK, Yousuf B, Bigelow PL, Eerd DV (2015) Effectiveness of health promotion programmes for truck drivers: a systematic review. Health Educ J 74(3):270–286. 10.1177/0017896914533953

[CR35] Nylund-Gibson K, Choi AY (2018) Ten frequently asked questions about latent class analysis. Transl Issues Psychol Sci 4(4):440–461. 10.1037/tps0000176

[CR36] Nylund-Gibson K, Grimm RP, Masyn KE (2019) Prediction from latent classes: a demonstration of different approaches to include distal outcomes in mixture models. Struct Equ Model 26(6):967–985. 10.1080/10705511.2019.1590146

[CR37] Nylund KL, Asparouhov T, Muthén BO (2007) Deciding on the number of classes in latent class analysis and growth mixture modeling: a Monte Carlo simulation study. Struct Equ Model 14(4):535–569. 10.1080/10705510701575396

[CR38] Oberski DL (2016) Beyond the number of classes: separating substantive from non-substantive dependence in latent class analysis. Adv Data Anal Classif 10(2):171–182. 10.1007/s11634-015-0211-0

[CR39] Patel AS, Moake TR (2023) Health behavior profiles: a person-centered approach to employee health. Soc Sci J 200:1–14. 10.1080/03623319.2023.2178441

[CR40] Petrovic D et al (2018) The contribution of health behaviors to socioeconomic inequalities in health: a systematic review. Prev Med 113:15–31. 10.1016/j.ypmed.2018.05.003 29752959 10.1016/j.ypmed.2018.05.003

[CR41] Prochaska JO, DiClemente CC (1983) Stages and processes of self-change of smoking: toward an integrative model of change. J Consult Clin Psychol 51(3):390–395. 10.1037/0022-006X.51.3.390 6863699 10.1037//0022-006x.51.3.390

[CR42] Prochaska JO, Spring B, Nigg CR (2008) Multiple health behavior change research: an introduction and overview. Prev Med 46(3):181–188. 10.1016/j.ypmed.2008.02.001 18319098 10.1016/j.ypmed.2008.02.001 PMC2288583

[CR43] Puhkala J et al (2015) Lifestyle counseling to reduce body weight and cardiometabolic risk factors among truck and bus drivers-a randomized controlled trial. Scand J Work Environ Health 41(1):54. 10.5271/sjweh.3463 25310464 10.5271/sjweh.3463

[CR44] Ratz T, Voelcker-Rehage C, Pischke CR, Muellmann S, Peters M, Lippke S (2021) Health-related lifestyle and dropout from a web-based physical activity intervention trial in older adults: a latent profile analysis. Health Psychol 40(8):481–490. 10.1037/hea0001091 34472906 10.1037/hea0001091

[CR45] Rhodes RE, Quinlan A, Mistry CD (2016) Do other goals influence physical activity? A systematic review examining the relationship between other goals and physical activity behavior. Prev Med 91:306–317. 10.1016/j.ypmed.2016.08.033 27568235 10.1016/j.ypmed.2016.08.033

[CR46] RIVM (2024) Gezondheidsenquête/Leefstijlmonitor. https://statline.rivm.nl/#/RIVM/nl/dataset/50080NED/table?dl=AF102

[CR47] Robroek SJ, van Lenthe FJ, van Empelen P, Burdorf A (2009) Determinants of participation in worksite health promotion programmes: a systematic review. Int J Behav Nutr Phys Act 6:26. 10.1186/1479-5868-6-26 19457246 10.1186/1479-5868-6-26 PMC2698926

[CR48] Rubio-Tomás T, Skouroliakou M, Ntountaniotis D (2022) Lockdown due to COVID-19 and its consequences on diet, physical activity, lifestyle, and other aspects of daily life worldwide: a narrative review. Int J Environ Res Public Health 19(11):6832. 10.3390/ijerph19116832 35682411 10.3390/ijerph19116832 PMC9180681

[CR49] Saint Onge JM, Krueger PM (2017) Health lifestyle behaviors among U.S. adults. SSM 3:89–98. 10.1016/j.ssmph.2016.12.009 10.1016/j.ssmph.2016.12.009 PMC5544030 28785602

[CR50] Schaap R, Schaafsma FG, Bosma AR, Huysmans MA, Boot CRL, Anema JR (2020) Improving the health of workers with a low socioeconomic position: intervention mapping as a useful method for adaptation of the participatory approach. BMC Public Health 20(1):1–13. 10.1186/s12889-020-09028-2 32560709 10.1186/s12889-020-09028-2 PMC7304135

[CR51] Schell C, Godinho A, Cunningham JA (2021) To thine own self, be true: examining change in self-reported alcohol measures over time as related to socially desirable responding bias among people with unhealthy alcohol use. Subst Abus 42(1):87–93. 10.1080/08897077.2019.1697998 32040383 10.1080/08897077.2019.1697998

[CR52] Schnittker J, Bacak V (2014) The increasing predictive validity of self-rated health. PLoS ONE 22(1):e84933. 10.1371/journal.pone.0084933 10.1371/journal.pone.0084933 PMC3899056 24465452

[CR53] Schreuder KJ, Roelen CA, Koopmans PC, Groothoff JW (2008) Job demands and health complaints in white and blue collar workers. Work 31(4):425–432 19127013

[CR54] Sinha P, Calfee CS, Delucchi KL (2021) Practitioner’s guide to latent class analysis: methodological considerations and common pitfalls. Crit Care Med 49(1):e63–e79. 10.1097/CCM.0000000000004710 33165028 10.1097/CCM.0000000000004710 PMC7746621

[CR55] Smit DJM, Proper KI, Engels JA, Campmans JMD, van Oostrom SH (2023) Barriers and facilitators for participation in workplace health promotion programs: results from peer-to-peer interviews among employees. Int Arch Occup Environ Health 96(3):389–400. 10.1007/s00420-022-01930-z 36305914 10.1007/s00420-022-01930-z PMC9614189

[CR56] Soler RE et al (2010) A systematic review of selected interventions for worksite health promotion. The assessment of health risks with feedback. Am J Prev Med 38:S237–S362. 10.1016/j.amepre.2009.10.030 20117610 10.1016/j.amepre.2009.10.030

[CR57] Sorensen G, Quintiliani L, Pereira L, Yang M, Stoddard A (2009) Work experiences and tobacco use: findings from the Gear Up for Health study. J Occup Environ Med 51(1):87–94. 10.1097/JOM.0b013e31818f69f8 19136877 10.1097/JOM.0b013e31818f69f8

[CR58] Sorensen G et al (2010) Tobacco use cessation and weight management among motor freight workers: results of the gear up for health study. Cancer Causes Control 21:2113–2122. 10.1007/s10552-010-9630-6 20725775 10.1007/s10552-010-9630-6 PMC3275135

[CR59] Spurk D, Hirschi A, Wang M, Valero D, Kauffeld S (2020) Latent profile analysis: a review and “how to” guide of its application within vocational behavior research. J Vocat Behav 120:103445. 10.1016/j.jvb.2020.103445

[CR60] STL (2024) Bronnen-en tabellenboek arbeidsmarktrapportage 2023 (Source and table book Labor market report 2022). STL, Gouda

[CR61] Stockwell S et al (2021) Changes in physical activity and sedentary behaviours from before to during the COVID-19 pandemic lockdown: a systematic review. BMJ Open Sport Exerc Med 7(1):e000960. 10.1136/bmjsem-2020-000960 10.1136/bmjsem-2020-000960 PMC7852071 34192010

[CR62] Sun F, Norman IJ, While AE (2013) Physical activity in older people: a systematic review. BMC Public Health 13:1–17. 10.1186/1471-2458-13-449 23648225 10.1186/1471-2458-13-449 PMC3651278

[CR63] Tcymbal A et al (2024) Validity, reliability, and readability of single-item and short physical activity questionnaires for use in surveillance: a systematic review. PLoS ONE 19(3):e0300003. 10.1371/journal.pone.0300003 38470871 10.1371/journal.pone.0300003 PMC10931432

[CR64] Tuomi K, Ilmarinen J, Jahkola A, Katajarinne L, Tulkki A (2003) Work ability index, 2nd edn, Helsinki

[CR65] UNESCO (2012) International Standard classification of Education ISCED 2011. UNESCO Institute for Statistics, Montreal

[CR66] USDA (2020) Dietary guidelines for Americans 2020–2025, 9th edn

[CR67] USDHHS (2018) Physical activity guidelines for Americans, 2nd edn

[CR68] Väisänen D et al (2023) Mediation of lifestyle-associated variables on the association between occupation and incident cardiovascular disease. Prev Med 167:107411. 10.1016/j.ypmed.2022.107411 36592676 10.1016/j.ypmed.2022.107411

[CR69] Van den Borre L, Deboosere P (2018) Investigating self-reported health by occupational group after a 10-year lag: results from the total Belgian workforce. Arch Public Health 76:68. 10.1186/s13690-018-0313-1 30455881 10.1186/s13690-018-0313-1 PMC6223069

[CR70] van der Beek AJ (2012) World at work: truck drivers. Occup Environ Med 69(4):291–295. 10.1136/oemed-2011-100342 22006936 10.1136/oemed-2011-100342

[CR71] Vermunt JK, Magidson J (2021) How to perform three-step latent class analysis in the presence of measurement non-invariance or differential item functioning. Struct Equ Model 28(3):356–364. 10.1080/10705511.2020.1818084

[CR72] Virgara R et al (2024) Keep on truckin’: how effective are health behaviour interventions on truck drivers’ health? A systematic review and meta-analysis. BMC Public Health 24(1):2623. 10.1186/s12889-024-19929-1 39334100 10.1186/s12889-024-19929-1 PMC11438120

[CR73] Ware JE, Kosinski M, Keller SD (1996) A 12-item short-form health survey: construction of scales and preliminary tests of reliability and validity. Med Care 34(3):220–233 8628042 10.1097/00005650-199603000-00003

[CR74] Weller BE, Bowen NK, Faubert SJ (2020) Latent class analysis: a guide to best practice. J Black Psychol 46(4):287–311. 10.1177/0095798420930932

[CR75] Wronska MD, Coffey M, Robins A (2022) Determinants of nutrition practice and food choice in UK construction workers. Health Promot Int 37(5):daac129. 10.1093/heapro/daac129 36166265 10.1093/heapro/daac129

[CR77] Wardle J, Steptoe A (2003) Socioeconomic differences in attitudes and beliefs about healthy lifestyles. J Epidemiol Community Health 57(6):440-3. 10.1136/jech.57.6.440 10.1136/jech.57.6.440 PMC1732468 12775791

[CR76] Xia N et al (2020) Patterns of cancer-related risk behaviors among construction workers in Hong Kong: a latent class analysis approach. Saf Health Work 11(1):26–32. 10.1016/j.shaw.2019.12.009 32206371 10.1016/j.shaw.2019.12.009 PMC7078528

